# Trends in the Use of Complementary and Alternative Therapies among US Adults with Current Asthma

**DOI:** 10.3390/epidemiologia4010010

**Published:** 2023-03-21

**Authors:** Chukwuemeka E. Ogbu, Chisa Oparanma, Stella C. Ogbu, Otobo I. Ujah, Menkeoma L. Okoli, Russell S. Kirby

**Affiliations:** 1 Chiles Center, College of Public Health, University of South Florida, Tampa, FL 33612, USA; 2 Department of Medicine, Kharkiv National Medical University, 61022 Kharkiv, Ukraine; 3 Department of Biomedical Science, Tulane University School of Medicine, New Orleans, LA 70112, USA; 4 Department of Internal Medicine, Christus Health, Texas A&M University, Longview, TX 75601, USA

**Keywords:** complementary and alternative therapies, asthma, herbs, breathing exercises, vitamins, acupuncture, yoga, trends

## Abstract

Complementary and Alternative Medicines/Therapies (CAM) are commonly used by US asthma adults, yet little is known about recent trends in their use. Our aim was to report trends in CAM use among US adults with current asthma. We conducted a serial cross-sectional study using nationally representative data from the BRFSS Asthma Call-Back Survey (ACBS) collected between 2008 and 2019 (sample size per cycle, 8222 to 14,227). The exposure was calendar time, as represented by ACBS cycle, while the main outcomes were use of at least one CAM and eleven alternative therapies. We analyzed CAM use overall and by population subgroups based on age, gender, race/ethnicity, income, and daytime and night-time asthma symptoms. Our findings show that there was an increase in the use of at least one CAM from 41.3% in 2008 to 47.9% in 2019 (*p*-trend < 0.001) and an upward trend in the use of herbs, aromatherapy, yoga, breathing exercises, homeopathy, and naturopathy (*p*-trend < 0.05). However, the use of vitamins, acupuncture, acupressure, reflexology, and other CAM therapies remained stable (*p*-trend > 0.05). These trends varied according to population characteristics (age, sex, race, income) and asthma symptoms. In conclusion, our study suggests that CAM use among US adults with current asthma is either increasing or stable, and further studies are needed to explore the factors influencing these trends.

## 1. Introduction

Asthma is a chronic condition that is marked by persistent inflammation of the airways [1]. It is characterized by recurrent respiratory symptoms, including wheezing, chest tightness, cough, and shortness of breath, which fluctuate in severity and frequency, and are accompanied by unpredictable expiratory airflow restriction [1]. An estimated 21 million adults and 4 million children are living with asthma in the United States, with 10 million (41%) of those affected having at least one episode of an asthma attack within the past 12 months [2]. With an increasing prevalence yearly, asthma is a significant health and economic burden to the patient, their family, and society. With no cure, asthma is a condition that remains poorly controlled in many people despite the availability of pharmacological interventions, evidence-based treatment guidelines, and multiple care pathways [3]. This has led to many people seeking non-pharmacological approaches to manage their asthma symptoms, even without their physician’s knowledge [4].

In a review conducted by Zollman and Vickers et al. (1999), the term complementary and alternative medicine (CAM) was defined as a comprehensive sphere of therapeutic resources that includes all healthcare systems, techniques, and methodologies, along with their associated beliefs and theories [5]. Patterns of CAM use differ according to age, sex, race, socioeconomic status, geography, and religious and spiritual backgrounds [6]. Younger age, female patients, white race, higher education, and income were characteristics associated with more use [6,7]. Several studies have identified why people seek complementary and alternative treatment. Vincent and Furnham (1996) conducted an empirical study that revealed the primary factors motivating patients to seek complementary treatment [8]. These included a favorable assessment of complementary medicine, the lack of efficacy of conventional treatment for their specific ailment, apprehension regarding the harmful side effects of conventional medicine, apprehensions about communication with physicians, and the accessibility of complementary medicine [8].

In essence, the prevalence of alternative medicine use is on the rise, with an estimated 75% of the US population admitting to CAM use at least once in their lifetime [9]. A review by Slader et al. (2006) estimated that 4–79% of adults with asthma use CAM [10]. However, they noted challenges in making conclusions due to poor methodology and little consistency among the available studies. Even though studies have shown varying degrees of benefit from their use [3,4,5,6,7,8,9,10,11,12,13,14,15,16,17,18,19,20,21,22], there is a need to acknowledge the potential for harm. CAM may directly harm patients due to poorly understood mechanisms of action, either as a result of potential toxicity, or as a result of improper practice or use [9]. Furthermore, CAM may interfere with other medications and reduce their efficacy, leading to worse outcomes [9]. Indirectly, the harm of CAM use may result from patients delaying or avoiding seeking proper asthma treatment, leading to worsening symptoms and overall quality of life [9]. Hence, there is a need to understand how these trends have changed over time as there is no current study, to the best of our knowledge, that has examined trends in CAM use among asthmatics in the last decade.

Complementary and alternative therapy consists of a wide range of disciplines, and new forms of therapy are continually being developed. The most used therapies in the treatment of asthma include herbs, homeopathy, acupuncture, yoga, and breathing exercises [7,9,10].

Herbs are historically one of the oldest forms of alternative treatment and were among the most conventional methods of treating ailments before the introduction of modern medicine. A review by Clarke et al. (2015) found that several herbal medicines have shown promising results in asthma patients, especially a group of herbs called the Anti-asthma Herbal Medicine Intervention (ASHMI) [11]. Similar studies also documented that the prevalence of herbal use in asthma treatment is increasing and shows promise for the future but is limited by a lack of quality clinical trials [12,13]. Homeopathy involves the use, in dilution, of substances that cause symptoms in their undiluted form [14]. It is one of the most widespread forms of alternative therapy for asthma. Even though little is understood about their mechanism of action, a systemic review concluded that their effects are holistic [14]. The treatment results also varied based on the type of homeopathy used, but they show that its use continues to grow in recent times [14]. Acupuncture is a traditional Chinese medical practice that involves the insertion of thin needles into various body parts [15]. This therapy regulates the balance of blood and body by stimulating acupuncture points of the human body to achieve the effect of balancing the Qi [15]. A systematic review by Chen et al. (2020) found that the frequency of randomized controlled trials (RCT) of acupuncture for asthma is rising. However, its evidence-based medicinal benefit is still lacking [16]. Traditional yoga is a group of physical, mental, and spiritual practices or disciplines to control and unite the mind, body, and spirit [17]. A meta-analysis involving RCTs concluded that asthma patients observed symptom relief through yoga, especially when combined with breathing exercises [17]. Breathing exercises are another documented therapy in the literature. Exercises that include hyperventilation reduction techniques can improve asthma symptoms or reduce inhaler medication use in adults with poorly controlled asthma without harmful effects [18,19].

Other CAM therapies include nutritional supplements (vitamins), acupressure, reflexology, naturopathy, and aromatherapy. Vitamin use has also grown tremendously, especially vitamins A, C, D, and E, possibly because of their potential immunomodulatory functions [9]. Studies have found nothing to support or deter their complementary therapy use [9]. Reflexology is a therapy that involves applying finger pressure to various areas of the body [20]. Its application in asthma management has also been increasing [7,10,20,21]. Acupressure is a kind of therapy that involves applying manual pressure to specific acupoints on the body [22]. Acupressure encourages the movement of life energy through the meridians inside the body [22]. There is a well-reported benefit when acupressure is combined with acupuncture and reflexology [15]. Due to a small number of clinical trials, a difference in clinical outcome in adults has not been established, highlighting the need for more clinical trials [22]. Naturopathy includes a wide range of natural treatments that help induce positive health and ease disease symptoms by acting at physical and mental levels [7]. Aromatherapy is a type of therapy that uses essential oils to achieve physical and mental well-being [7,10].

Given the increase in complementary and alternative therapy use from prior studies, there are no recent studies that have evaluated their trends among patients with asthma in the United States. Our study aims to fill this gap by analyzing recent US population-based data available over the years (2008 to 2019) for all CAM methods. We also examined trends in the use of these CAM methods by age, sex, race, income status, and asthma impairment symptoms (daytime and nighttime symptoms).

## 2. Materials and Methods

### 2.1. Study Population

For this trend analysis, we utilized the Adult Asthma Call-back Survey (ACBS) from the Behavioral Risk Factor Surveillance System (BRFSS) between 2008 and 2019. The BRFSS ACBS is a cross-sectional study that examines non-institutionalized American adults aged 18 years and older. BRFSS is a state-based monitoring system overseen by the CDC which collects information on healthcare access and utilization, preventive health practices, health risk behaviors, chronic disease, preventable infectious disease, and injuries. The ACBS, which is a follow-up survey conducted two weeks after the BRFSS, provides yearly data on asthma, including demographics and disease histories. It aims to address critical questions regarding the health and experiences of people with asthma and generates data at both the local and state level. Participants in the BRFSS who reported a history of asthma diagnosis are eligible to participate in the ACBS and are chosen randomly.

To assess the prevalence of asthma in the BRFSS, two questions were employed. An affirmative response to the questions “Have you ever been told by a doctor, nurse, or other healthcare professional that you have asthma?” and “Do you currently have asthma?” were used to indicate lifetime asthma and current asthma status, respectively. Responding affirmatively to the first question was a requirement for participating in the follow-up ACBS conducted over the telephone. Response rates for the ACBS were computed according to the Council of American Survey and Research Organizations guidelines. Among the 31 states and the District of Columbia that participated in the 2008-to-2019 survey, the median ACBS response rate ranged from 36.9% to 60.0% [23].

Our study sample included 160,285 adults who reported a history of asthma, of which 125,978 were adults who presently had asthma between 2008 and 2019. The ACBS received approval from the Institutional Review Boards of the states and the Ethics Review Board of the Asthma and Community Health Branch in the National Center for Environmental Health, and all participants gave informed consent. As the ACBS is a publicly available dataset, this study is exempt from full institutional review board review. Additional information on the data, sampling methodology, and analytical protocols can be found elsewhere [24].

#### 2.1.1. Complementary and Alternative Therapy Use Ascertainment

ACBS participants were first asked if they used any complementary medicine or alternative therapies to control their asthma. The survey item “In the past 12 months, have you used to control asthma?” was used to ascertain use of non-traditional, complementary, and alternative healthcare or asthma treatments. The eleven alternative treatments assessed in this manner were herbs, vitamins, acupuncture, acupressure, aromatherapy, homeopathy, reflexology, yoga, breathing techniques, naturopathy, and other therapies. Responses were recorded as “Yes” or “No”. We created a variable to ascertain the use of at least one CAM method (“Ever use of CAM”). Any participant who recorded a yes response in the use of any of the eleven therapy categories were reported as having ever used CAM to control their asthma.

#### 2.1.2. Covariates Ascertainment

We used surrogate measures of asthma control/severity, namely daytime symptoms (categorized as ≤2 days a week, >2 days a week, throughout the day) and night-time symptoms (categorized as ≤2 days a week, 1–3 times a week, ≥4 times a week), due to the ACBS lacking some necessary clinical measures for current impairment (such as pulmonary function measures) and future risk assessment (asthma exacerbations or progressive decline in lung function). This adaptation was based on the 2007 National Asthma Education and Prevention Program Expert Panel Report 3 Guidelines (NAEPP), and prior studies using the ACBS have also adopted this modified NAEPP guidelines for assessing asthma control. [25,26,27].

The other covariates in the analysis were categorized as follows: age (<55 years or ≥55 years), sex (male versus female), smoking status (current smoker versus former/never a smoker), race (white non-Hispanic, black, Hispanic, or multiracial), and income (<$25,000/year or ≥$25,000/year).

### 2.2. Statistical Analysis

To improve the representativeness of the US adult population, the Adult ACBS utilizes a complex, multistage stratified survey design. In accordance with analytical guidelines recommended by the ACBS, complex analytical procedures were employed, and appropriate weighting was applied to adjust for sampling strata, clusters, and primary sampling unit (PSU).

The prevalence of ever using CAM was assessed within each ACBS cycle and evaluated the statistical significance of trends using a two-sided test at the α  = 0.05 level, adjusted for age and sex. The prevalence ratios for CAM use in 2019 vs. 2008 were calculated and the absolute difference in prevalence was presented. Additionally, trends of individual therapies were explored and reported with their corresponding statistical significance, adjusted for age and sex. Results were presented as a decrease (prevalence ratio < 1, *p* for trend < 0.05), increase (prevalence ratio > 1, *p* for trend < 0.05), or stable (*p* for trend ≥ 0.05) trend. Weighted logistic regression was used for the analysis and appropriate weighting was applied to account for the sampling strata, cluster, and primary sampling unit (PSU) in accordance with the ACBS guidelines for complex survey design.

We examined trends in ever using CAM and individual therapies by presenting results in graphs and tables stratified by age (<55 years or ≥55 years), sex (male vs. female), daytime symptoms (≤2 days a week, >2 days a week, throughout the day), night-time symptoms (≤2 days a week, 1–3 times a week, ≥4 times a week), income (<$25,000/year or ≥$25,000/year and above), and race (white non-Hispanic, black non-Hispanic, Hispanic, and multiracial/other race).

Sensitivity analyses were conducted for ever using CAT and for each individual alternative therapy by examining quadratic trends. To address the consistency of trends over the study period, we re-ran the model with both linear and quadratic time variables (age and sex). If quadratic changes were detected, we conducted Joinpoint regression analyses and calculated the annual percentage change before and after potential inflection points using Joinpoint Regression Program Software (National Cancer Institute, Bethesda, MD, USA), version 4.2.0 [28]. All analyses were performed using SAS software version 9.4 (SAS Institute Inc., Cary, NC, USA) and Microsoft Data Analysis ToolPak for Excel 2016 version. Results were presented in tables and graphs.

## 3. Results

### 3.1. Use of any CAM among US Adults with Current Asthma in 2019

In 2019, 48% (95% CI, 46–50%) of US adults with active asthma reported using any CAM in the preceding 12 months (Table 1).

The use of at least one CAM was found to be associated with several sociodemographic and asthma symptom variables. Adults aged 18–55 years were more likely to report ever using CAM compared to those aged 55 years or older, with 52% (95% CI, 49–55%) and 42% (95% CI, 39–44%) reporting use, respectively. Females (53% [95% CI, 50–55%]) were more likely to use at least one CAM than males (40% [95% CI, 36–44%]). The multiracial/other race non-Hispanic group had the highest prevalence of ever using CAM at 55% (95% CI, 46–65%), while white non-Hispanic adults had the lowest at 46% (95% CI, 44–49%). Prevalence was higher in adults in <$25,000 income level (54% [95% CI, 47–60%]) than those in the ≥$25,000 income level (45% [95% CI, 42–48%]). By asthma symptoms, use of at least one CAM was highest among adults with daytime symptoms >2 days/week (59% [95% CI, 55–63]) and lowest in those with daytime symptoms throughout the day (43 (95% CI, 40–46]). More so, adults with 1–3 times/week night-time symptoms had the highest prevalence (59% [95% CI, 51–68%]) while those with ≥4 times/week symptoms had the lowest (44% [95% CI, 41–47%]).

### 3.2. CAM Trend across Years

Overall, there was increased use of at least one CAM, with 41.3% reporting use in 2008 and 47.9% reporting used in 2019 (difference, 5.6% [95% CI, 3.5% to 9.7%]) (Table 2).

**Figure 1:**
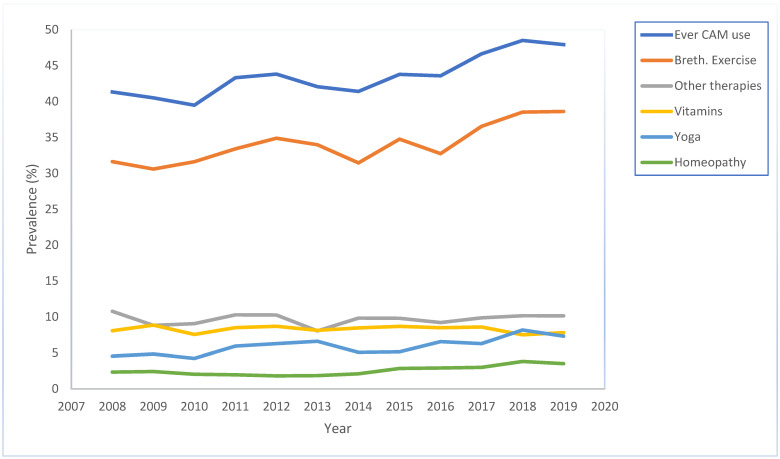
Trend in use of atleast one CAM, breathing exercise, other CAM therapies, vitamins, yoga, and homeopathy among adults with current asthma, 2008 through 2019.

For each individual therapy use, there was a change in the use of herbs, with 8.05% reporting use in 2019 (difference, 1.6% [95% CI, −0.13% to 3.3%]). Use of aromatherapy (difference, 5.7% [95% CI, 3.8% to 7.6%]), homeopathy (difference, 1.2% [95% CI, 0.2% to 2.2%]), yoga (difference, 2.8% [95% CI, 0.1% to 4.5%]), breathing exercises (difference, 7.0% [95% CI, 3.9% to 10.1%]), and naturopathy (difference, 0.6% [95% CI, −0.2% to 1.4%]) also increased between 2008 and 2019 (*p*-trend < 0.05, PR > 1) (Figure 1).

**Figure 2:**
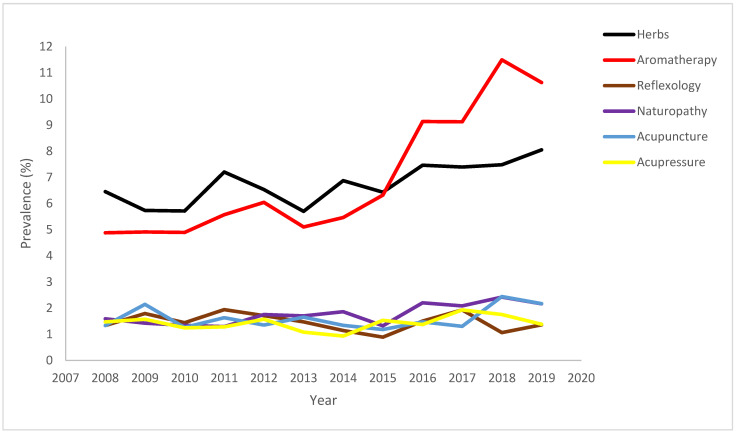
Trend in use of herbs, aromatherapy, reflexology, naturopathy, acupuncture, and acu-pressure among adults with current asthma, 2008 through 2019.

The trend in use of vitamins (difference, −0.3% [95% CI, −2.0% to 1.5%]), acupuncture (difference, 0.8% [95% CI, −0.2% to 0.02%]), acupressure (difference, −0.09% [95% CI, −0.8% to 0.6%]), reflexology (difference, 0.02% [95% CI, −0.6% to 0.6%]), and other alternative therapy (difference, −0.6% [95% CI, −2.4% to 1.1%]) was stable (*p* > 0.05) (Figure 2).

### 3.3. Trend by Subgroups

Trends by age, sex, race/ethnicity, income, and daytime and night-time asthma symptoms were also evaluated (Table S1 in the Supplement). Use of any CAM increased among adults aged 18–55 years and in adults 55 years or older (Figure S1 in the Supplement). Although women reported more CAM use than men, trends increased in both groups. Use of any CAM among non-Hispanic white adults increased between 2008 and 2019 (*p* < 0.05; difference, 6.7% [95% CI, 3.5% to 9.9%]), remained stable among multiracial/other (*p* > 0.05) and Hispanic adults (*p* > 0.05) but decreased among non-Hispanic black adults (*p* < 0.05; difference, −3.98% [95% CI, −16.2% to 8.2%]). Trends in ever using CAM increased among adults with ≤2 days/week daytime symptoms (difference, 9.82% [95% CI, 1.3% to 18.3%]), >2 days/week daytime symptoms (difference, 10.2% [95% CI, 4.6% to 15.8%]), and daytime symptoms throughout the day (difference, 5.7% [95% CI, 1.7% to 9.7%]). There was also an increasing trend among adults with ≤2 times/month night-time symptoms (difference, 2.2% [95% CI, −8.7% to 13.1%]), 1–3 times/week night-time symptoms (difference, 14.2% [95% CI, 6.8% to 21.5%]), and ≥4 times/week night-time symptoms (difference, 6.6% [95% CI, 3.1% to 10.2%]).

There was an increasing trend in the use of herbs, aromatherapy, breathing exercises, homeopathy, and naturopathy in adults aged 18–55 years and ≥55 years, while trends in vitamins and acupuncture remained stable in both age categories (Table S1). The trend in the use of aromatherapy (difference,6.6% [95% CI, 4.5% to 8.6%]), yoga (difference, 2.2% [95% CI, −0.04% to 4.5%]), naturopathy (difference, 0.2% [95% CI, −0.13% to 1.29%]), homeopathy (difference, 1.0% [95% CI, −0.05% to 2.1%] and breathing exercises (difference, 5.8% [95% CI, 2.0% to 9.6%]) increased in adults with income >$25,000. In contrast, vitamin, acupuncture, acupressure, reflexology trends, and other alternative therapy use remained stable in adults with incomes <$25,000 and >$25,000 (Table S1).

Use of herbs, aromatherapy, Yoga, breathing exercises, and homeopathy increased in adults with daytime symptoms ≤2 days/week, >2 days/week, and throughout the day, while reflexology and vitamins were stable in the three daytime symptom groups (Table S1). Daytime symptoms noted some mixed trend patterns: use of naturopathy decreased in those with ≤2 days/week daytime symptoms and increased in adults with >2 days/week and throughout the day, acupuncture and acupressure use increased in adults with ≤2 days/week and remained stable in those with >2 days/week and throughout the day, and use of other CAM therapy increased in those with >2 days/week and remained stable in adults with ≤2 days/week and throughout the day (Table S1).

The use of herbs, aromatherapy, yoga, homeopathy, naturopathy, and other CAM therapies increased amongst non-Hispanic white adults. At the same time, there were stable trends in herbal, aromatherapy, yoga, breathing exercises, homeopathy, naturopathy, acupuncture, acupressure, reflexology, and other CAM therapies among Hispanics (Table S1 in Supplement).

## 4. Discussion

In this extensive, nationally representative survey of US adults with active asthma, the use of at least one CAM increased from 41.3% in 2008 to 47.9% in 2019. The use of each individual complementary/alternative therapy showed increasing or stable trends. Trends for using at least one CAM and individual alternative therapies varied across age, sex, asthma day symptoms, night symptoms, and income.

The varying prevalence of CAM use among adults with active asthma agrees with earlier studies reporting that CAM use rates range from 4% to 79% [29]. In our study, the increasing trend seen among users of any CAM is largely driven by increasing trends in some of the individual therapies, principally herbal, aromatherapy, homeopathy, yoga, breathing exercises, and naturopathy across the 12-year survey cycle.

Individual therapies, such as herbs, aromatherapy, homeopathy, yoga, breathing exercise, and naturopathy, showed increasing trends across the 12-year cycle. This increasing trend may reflect the overall positive valuation of complementary and alternative treatment, concern about the adverse effects of pharmaceutical medicines, and availability of these modalities in contrast to pharmaceutical medicines [8]. Increasing trends in herbal use, for example, could stem from evidence from clinical trials. For example, a 4-week double-blind, randomized trial compared oral doses of Anti-asthma Herbal Medicine Intervention (ASHMI) with prednisone and found that although ASHMI showed significantly fewer improvements in lung function compared to prednisone, it still displayed clinically relevant reductions in patient symptom scores and enhancements in lung function, suggesting potential use among patients with less severe forms of asthma [11]. Additionally, although yoga cannot be considered a routine intervention for asthmatic patients, it may be considered an ancillary alternative to breathing exercises for asthma patients [17]. The increasing trends in CAM use could also be due to changes in treatment guidelines, such as international guidelines for the management of asthma that include the option of breathing exercise programs as an adjuvant to pharmacological treatment [1]. We found stabilization of the trends in using vitamins, acupuncture, acupressure, reflexology, and other alternative therapies across all-year cycles. The stable trend seen in vitamin use may reflect the increased scrutiny of multivitamins and minerals following studies showing no benefit [30] and the stance/recommendations of several professional bodies that concluded that there was insufficient or no evidence to support their use [31,32]. Furthermore, weaker evidence for the use of acupuncture [16], acupressure [22], and reflexology [20] could deter further use, especially in the current explosion in accessible health information.

Our study considered potential heterogeneity by population subgroups, and we examined trends by age, gender, daytime asthma symptoms, night-time asthma symptoms, and income. The use of at least one CAM increased in younger and older adults and was driven majorly by herbs, aromatherapy, yoga, breathing exercises, homeopathy, and naturopathy, whose trends also increased in the two age categories. Our findings agree with a prior study that found increased use of mind–body therapies among US adults aged 18 and above that participated in the National Health Interview Study (NHIS) between 2002 and 2007 [33]. Barnes et al. (2008) reported increased prevalence in the use of deep breathing exercises, yoga, and naturopathy in the general US adult population [33]. The general trend seen among our asthma population likely corresponds to general trends seen among the US population for these therapies.

Our observation of an increasing trend in ever using CAM, aromatherapy, homeopathy, breathing exercises, and yoga among those with higher income is consistent with a study that evaluated the prevalence of CAM use among chronic disease (prostate cancer patients) and reported a wide range of use with increasing trends in those with higher income [34,35]. We also found increasing trends in the use of naturopathy and homeopathy in the lower-income group and stable trends in vitamin, acupuncture, acupressure, reflexology, and other alternative therapy use in both adults with income <$25,000 and >$25,000, which highlights similar findings in the extant literature of the less consistent trends between CAM use and income [36].

In this study, there was an increasing trend in using seven of the eleven alternative therapies in females. Our study agrees with other population-based studies that found women more likely than men to use CAM, with the most prominent sex differentials seen in mind–body therapies [33,37]. A similar study also found that CAM use was higher in women with or without chronic diseases than in men [38]. The reason for this continuously increasing trend remains to be determined. Some explanations proffered include women being more open to the utilization of health services [39] and increased health-seeking behaviors of females compared to males [40]. Some researchers have suggested modern society’s progressive and dynamic nature, which supports women’s independence and personal transformation through self-reflection and self-discovery [41]. However, further longitudinal studies are needed to understand why these differences by gender exist across all spectrums of CAM use in patients with disease states.

We found mixed trends in CAM use by daytime and night-time asthma symptoms, irrespective of symptom severity. For instance, we found an increasing trend in herbal use in those with >2 days/week of daytime symptoms (moderate symptoms). Similarly, there was an increasing trend in aromatherapy use in those with ≤2 days/week daytime symptoms (mild symptoms). The reason for these mixed trends in patients with different asthma severity is unclear. While the role of health status and the ineffectiveness of a patient’s current treatment have been emphasized as a reason for using alternative therapies [8], these findings have not been consistent across studies [35,36]. Bishop et al. (2010) reported the use of CAM to be more common only in 21 out of their 55 studies in people with poor disease severity, providing evidence against the stereotypical belief that CAM users have more severe diseases or symptoms [36]. The inconsistent findings regarding trends in CAM use and asthma symptom severity might also be partly due to the cross-sectional study designs and cohort effects necessitating further prospective studies to understand this relationship.

Trends in CAM use among US adults with asthma varied by race/ethnicity. We found that the use of any CAM increased among white adults, decreased amongst black non-Hispanics, and had a stable trend among Hispanic and multi-and/or other non-Hispanic adults. The increasing trend continued among white non-Hispanics in herbal, aromatherapy, yoga, homeopathy, naturopathy, and other CAM therapies. These findings are similar to what has been reported in prior studies. Su and Li et al. (2011) reported that non-Hispanic white adults used at least one CAM the most, followed by Asians, African/black Americans, and Hispanics [42]. Surprisingly, we found increasing trends among black non-Hispanics in the use of herbs, breathing exercises, naturopathy, and acupuncture and stable trends among Hispanics in most of the eleven CAM modalities. The reason for these findings are unclear, as research has documented decreased use of these modalities of CAM among black non-Hispanic adults [43]. The stable trend among Hispanics in our study is also different from prior studies that reported increased CAM use due to limited access to conventional medicine [43]. Further studies to understand the drivers of these trends are warranted.

Our study has important strengths. The alternative therapies in the ACBS were collected from a nationally representative sample of non-institutionalized US adults, allowing for accurate estimation of CAM use in diverse population subgroups. The large sample size also facilitated the investigation of the trends of CAM use and other self-reported health characteristics, such as symptoms of asthma control and income. Using the ACBS data also allowed for examining CAM as individual therapy. CAM use is a heterogenous behavior with varying effectiveness, intensity, and side effects, and combining them all into an overall CAM cluster would lead to a loss in the direction of the effect of specific therapies. Exploring specific CAM therapies allowed us to parse specific trends among adults with active asthma. Moreso, the ACBS data were collected as part of a continuous national survey, allowing for the evaluation of trends over 12 years.

There are also limitations to note in this study. The BRFSS is a cross-sectional study; hence, temporality between asthma severity and alternative therapy use cannot be determined. Further, there may be selection bias; the BRFSS excludes the institutionalized and hospitalized population, making it possible to underestimate adults with active and severe asthma. Selection bias leads to disproportionately more healthy people than are in the general population and more participants that could access alternative therapies, causing a bias toward the null. In addition, asthma symptom status and CAM use were self-reported, resulting in recall bias and potential misclassification. Finally, ACBS did not include information on the dose and frequency of CAM use, making it difficult to determine the intensity of alternative therapy behaviors.

## 5. Conclusions

The overall use of at least one CAM among adults in the United States increased from 2008 to 2019, with individual CAM use either increasing or remaining stable during this period. However, trends in the use of individual therapies varied and were heterogeneous based on population characteristics and asthma daytime and night-time symptoms. Healthcare providers should be aware of these trends and perform medication/treatment reconciliation, particularly when patients are also taking asthma medications, to avoid potential side effects and interactions. Inquiring about CAM use in a non-judgmental way and attempting to comprehend the underlying motivations and actions is recommended for physicians and primary care providers. To grasp the causes and potential benefits behind the rising CAM usage trends, additional research is required.

## Figures and Tables

**Table 1:** Use of any CAM in prior 12 months among US adults with current asthma by population characteristics, 2019.

		Use of any CAM 2019		
	No. of Participants	No. of Participants	Weighted% (95% CI) ^a^	*p*-Value
Use of at least one CAM	8928	3946	47.92 (45.59–50.24)	
Age				<0.0001
18–55 years	3433	1702	51.75 (48.51–54.99)	
>55 years	5495	2244	41.64 (38.89–44.39)	
Sex				<0.0001
Women	5967	2808	52.58 (49.69–55.47)	
Men	2961	1138	40.03 (36.28–43.78)	
Race				0.16
White Non-Hispanic	7161	3057	46.04 (43.57–48.50)	
Black Non-Hispanic	417	188	48.88 (39.10–58.67)	
Multiracial/other race Non-Hispanic	623	326	55.32 (45.87–64.77)	
Hispanic	601	314	52.79 (45.02–60.56)	
Income				0.02
≥$25,000	5277	2228	44.93 (41.93–47.92)	
<$25,000	1096	573	53.51 (47.19–59.83)	
Daytime Symptoms				<0.0001
≤2 days/week	5532	2184	58.2 (50.96–65.43)	
>2 days/week	2305	1207	59.09 (54.95–63.22)	
Throughout the day	880	487	42.75 (39.78–45.71)	
Night symptoms				<0.0001
≤2 times a month	6917	2790	59.19 (50.70–67.68)	
1–3 times a week	1175	697	65.46 (60.15–70.77)	
≥4 times a week	578	344	43.94 (41.27–46.60)	

^a^ Data were weighted to be nationally representative.

**Table 2:** Trends in CAM use among US adults with current asthma, 2008 through 2019.

12-Month Prevalence of Use, Weighted%													2019 vs. 2008	
	2008	2009	2010	2011	2012	2013	2014	2015	2016	2017	2018	2019	PR (95% CI)	PD (95% CI)
Increased CAM use ^a^														
Ever using CAM	41.3	40.5	39.5	43.3	43.8	42.1	41.4	43.8	43.6	46.6	48.5	47.9	1.3 (1.2, 1.5)	6.6 (3.5, 9.7)
Herbs	6.5	5.7	5.7	7.2	6.5	5.7	6.9	6.4	7.5	7.4	7.5	8.1	1.3 (1.0, 1.6)	1.6 (−0.1, 3.3)
Aromatherapy	4.9	4.9	4.9	5.6	6.0	5.1	5.5	6.3	9.1	9.1	11.5	10.6	2.3 (1.7, 3.1)	5.7 (3.8, 7.6)
Homeopathy	2.3	2.4	2.0	2.0	1.8	1.9	2.1	2.9	2.9	3.0	3.8	3.5	1.5 (1.1, 2.2)	1.2 (0.2, 2.2)
Yoga	4.6	4.9	4.2	6.0	6.3	6.6	5.1	5.2	6.6	6.3	8.2	7.3	1.7 (1.2, 2.2)	2.8 (0.1, 4.5)
Breath. Exercises	31.6	30.6	31.6	33.4	34.9	34.0	31.5	34.8	32.7	36.5	38.5	38.6	1.4 (1.2, 1.6)	7.0 (3.9, 10.1)
Naturopathy	1.6	1.4	1.3	1.3	1.8	1.7	1.9	1.3	2.2	2.1	2.4	2.2	1.4 (0.9, 2.1)	0.6 (−0.2, 1.4)
Stable CAM use ^b^														
Vitamins	8.1	8.9	7.6	8.5	8.7	8.1	8.5	8.7	8.5	8.6	7.5	7.8	1.0 (0.8, 1.2)	−0.3 (−2.0, 1.5)
Acupuncture	1.3	2.1	1.3	1.6	1.4	1.7	1.3	1.2	1.5	1.3	2.4	2.2	1.6 (1.0, 2.8)	0.9 (−0.2, 0.1)
Acupressure	1.5	1.6	1.2	1.3	1.6	1.1	0.9	1.5	1.4	1.9	1.8	1.4	0.9 (0.6, 1.5)	−0.1 (−0.8, 0.6)
Reflexology	1.3	1.8	1.4	1.9	1.7	1.5	1.1	0.9	1.5	1.9	1.1	1.4	1.0 (0.7, 1.6)	0.02 (−0.6, 0.6)
Other therapies	10.8	8.8	9.1	10.3	10.3	8.1	9.8	9.8	9.2	9.9	10.2	10.2	0.9 (0.8, 1.1)	−0.6 (−2.4, 1.1)

CAM, Complementary and Alternative Medicine/therapies; PR, Prevalence Ratio; PD, Prevalence Difference, ^a^ Prevalence ratio > 1 and *p* for trend < 0.05, ^b^ *p* for trend ≥ 0.05.

## Data Availability

The data used to generate the findings of this study are publicly available in the CDC Asthma Call-Back Survey Website available at: https://www.cdc.gov/brfss/acbs/index.htm (accessed on 1 January 2023). The Asthma Call-back Survey (ACBS) is a product of CDC’s National Asthma Control Program (NACP).

## Supplementary Materials

The following supporting information can be downloaded at: https://www.mdpi.com/article/10.3390/epidemiologia4010010/s1, Figure S1: Trend in the use of at least one CAM by Day symptoms, Night symptoms, Age, Gender, Income and Race; Table S1: Trends in CAM use Among US Adults with active asthma, 2008 through 2019 by Age, Sex, Race, Income, Daytime and Nighttime symptoms.
