# Supplementary material for: Trends in the Use of Complementary and Alternative Therapies among US Adults with Current Asthma
Source: Epidemiologia (Basel). 2023 Mar 21;4(1):94–105. doi: 10.3390/epidemiologia4010010 (PMC10048134; doi:10.3390/epidemiologia4010010)
Supplement: Supplementary file 1 [file epidemiologia-04-00010-s001.zip › epidemiologia-2174428-supplementary figure.pdf]

Figure S1: Trend in the use of at least one CAM by Day symptoms, Night symptoms, Age, Gender, Income and Race

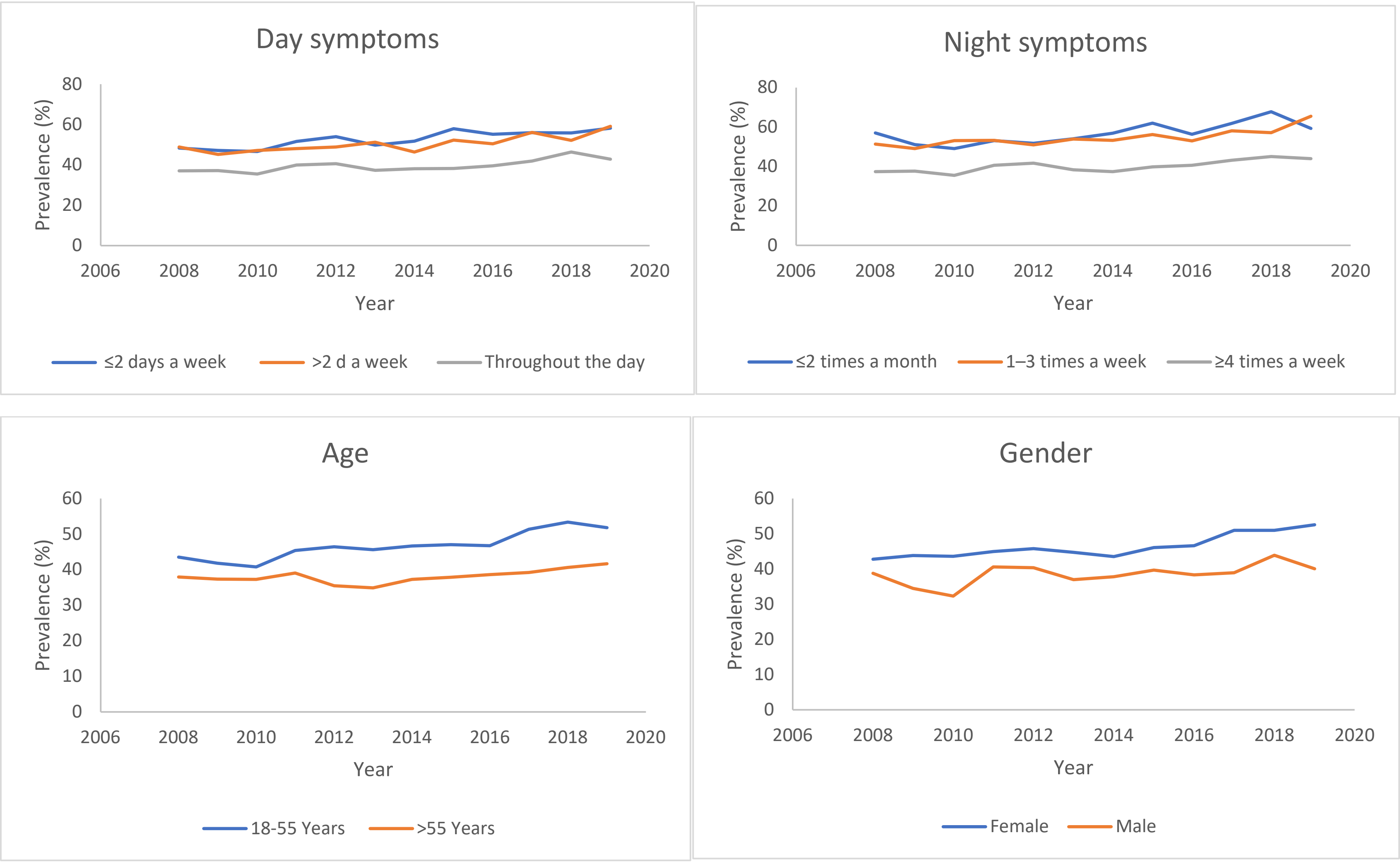

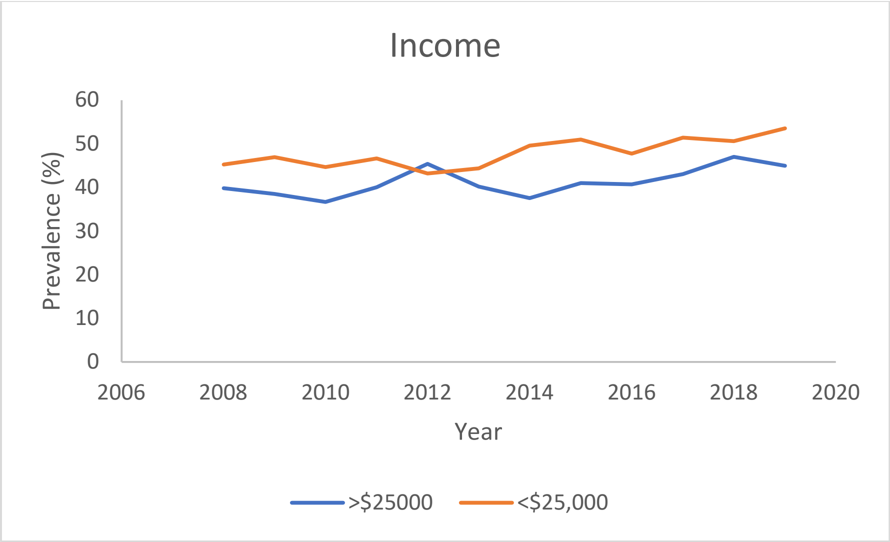
Income
Prevalence (%)
60
50
40
30
20
10
0
2006
2008
2010
2012
2014
2016
2018
2020
Year
>$25000
<$25,000

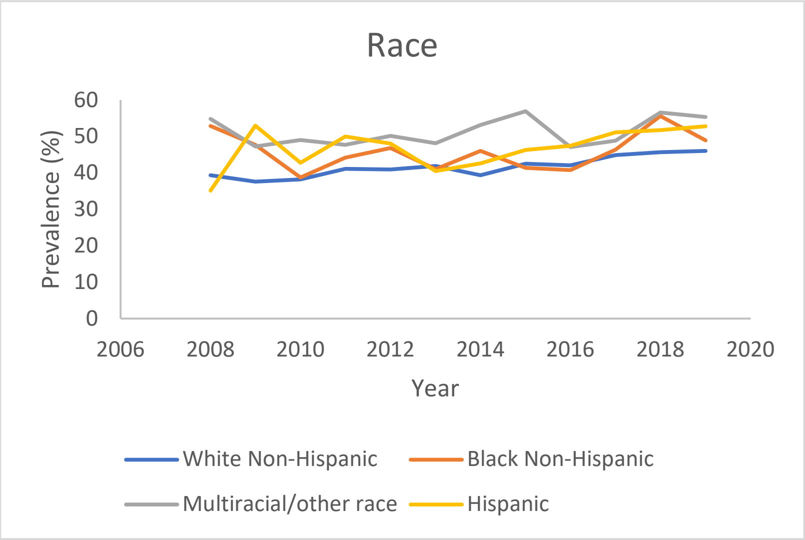
Race
Prevalence (%)
60
50
40
30
20
10
0
2006
2008
2010
2012
2014
2016
2018
2020
Year
White Non-Hispanic
Black Non-Hispanic
Multiracial/other race
Hispanic
