# Supplementary material for: Trends in the Use of Complementary and Alternative Therapies among US Adults with Current Asthma
Source: Epidemiologia (Basel). 2023 Mar 21;4(1):94–105. doi: 10.3390/epidemiologia4010010 (PMC10048134; doi:10.3390/epidemiologia4010010)
Supplement: Supplementary file 1 [file epidemiologia-04-00010-s001.zip › epidemiologia-2174428-supplementary.pdf]

Table S1: Trends in CAM use Among US Adults with active asthma, 2008 through 2019 by Age, Sex, Race, Income, Daytime and Nighttime symptoms

	ACBS 2008 (%)*	2009 (%)*	ACBS (%)*	ACBS 2011 (%)*	ACBS 2012(%)*	ACBS 2013(%)*	ACBS 2014(%)*	ACBS 2015(%)*	ACBS 2016(%)*	ACBS 2017(%)*	ACBS 2018(%)*	ACBS 2019(%)*	P-trend	2019 vs 2008	
**Ever use of CAM**														**Prevalence Ratio**	**Difference (%)**
**Day symptoms**															
≤2 days/week	48.37	47.14	46.61	51.67	54.01	49.71	51.68	57.93	55.12	55.91	55.81	58.2	0.011	1.20(1.02-1.39)	9.82(1.34-18.31)
>2 days/ week	48.87	45.21	47.15	48.09	48.79	51.23	46.33	52.23	50.37	56.07	52.16	59.09	<.0001	1.21(1.08-1.33)	10.21(4.61-15.82)
Throughout the day	37.01	37.21	35.53	39.95	40.51	37.32	38.13	38.26	39.57	41.88	46.38	42.75	0.0003	1.16(1.04-1.27)	5.74(1.74-9.74)
**Night symptoms**															
≤2 times/ month	56.99	51.04	49.09	53.02	51.65	53.98	56.78	61.84	56.2	61.73	67.74	59.19	0.0001	1.04(0.85-1.23)	2.20(-8.68 -13.08)
1–3 times/ week	51.28	49.01	52.97	53.22	50.9	53.8	53.18	56.15	52.96	58.05	57.02	65.46	0.0031	1.28(1.11-1.44)	14.18 (6.83-21.54)
≥4 times/week	37.32	37.61	35.54	40.51	41.6	38.29	37.32	39.79	40.5	43.07	45.09	43.94	<.0001	1.18(1.08-1.28)	6.61(3.08-10.15)
**Age**															
18-55 Years	43.49	41.77	40.78	45.35	46.44	45.56	46.63	47.04	46.69	51.32	53.4	51.75	<.0001	1.19(1.08-1.30)	8.25 (3.71-12.80)
>55 Years	37.89	37.3	37.27	39.01	35.45	34.92	37.26	37.82	38.58	39.18	40.6	41.64	0.007	1.10(0.99-1.20)	3.75(0.022-7.47)
**Gender**															
Female	42.8	43.78	43.55	44.93	45.78	44.72	43.47	46.03	46.56	50.93	50.99	52.58	<.0001	1.23(1.14-1.32)	9.78(6.17-13.39)
Male	38.73	34.45	32.33	40.59	40.32	36.94	37.77	39.67	38.3	38.88	43.95	40.03	0.0022	1.03(0.89-1.18)	1.3(-4.23 - 6.8)
**Income**															
>$25000	39.77	38.46	36.69	39.99	45.37	40.13	37.54	40.97	40.62	42.99	47.02	44.93	<.0001	1.13(1.03-1.23)	1.99(1.26-9.05)
<$25,000	45.17	46.89	44.61	46.56	43.21	44.32	49.5	50.9	47.67	51.37	50.57	53.51	0.0428	1.18(0.99-1.38)	8.34(0.35-16.3)
**Race**															
White Non-Hispanic	39.37	37.59	38.19	41.1	40.93	41.92	39.34	42.45	42.06	44.89	45.64	46.04	<.0001	1.17(1.08-1.26)	6.67(3.47-9.86)
Black Non-Hispanic	52.86	47.64	38.76	44.14	46.76	41.03	46.02	41.31	40.75	46.36	55.6	48.88	0.0163	0.92(0.70-1.15)	-3.98 (-16.21-8.24)
Multiracial/other race	54.75	47.21	49	47.65	50.09	48.1	53.1	56.89	47.06	48.79	56.55	55.32	0.0611	1.01(0.78 -1.24)	0.56(-11.97 - 13.09)
Hispanic	35.13	53.02	42.74	49.96	48.04	40.44	42.55	46.31	47.42	51.12	51.74	52.79	0.4978	1.50(1.09-1.91)	17.66(6.48 -28.85)
**Use of herbs**															
**Day symptoms**															
≤2 days/week	10.09	7.87	6.61	9.35	11.31	7.05	11.32	7.03	10.47	10.61	12.31	10.36	0.0511	1.03(0.57-1.48)	0.27(-4.27 -4.81)
>2 days/ week	8.74	7.15	6.34	8.22	7.47	7.4	8.05	7.99	9.1	11.36	7.83	12.32	0.0025	1.41(0.89-1.93)	3.59(-0.50 -7.67)
Throughout the day	4.97	4.69	5.39	6.51	5.51	4.97	5.59	5.73	6.43	5.41	6.77	6.27	0.2996	1.26(0.78-1.74)	1.30(-0.78-3.38)
**Night symptom**															
≤2 times/ month	12.13	10.57	6.82	7.67	15.66	11.97	13.3	12.39	12.3	15.03	12.73	11.8	0.0549	0.97(0.45 -1.50)	-0.34(-6.77 - 6.10)

1–3 times/ week	9.87	7.33	10.53	10.49	7.48	9.65	10.61	9.2	11.03	11.44	11.07	17.69	0.0055	1.79(1.06-2.52)	7.82(1.99-13.64)
≥4 times/week	5.19	4.91	4.64	6.45	5.37	4.33	5.46	5.39	6.38	6.05	6.19	6	0.0952	1.16(0.77-1.54)	0.82(-1.02 - 2.65)
**Age**															
18-55 Years	7.28	6.55	6.34	8.05	6.39	5.72	7.03	6.78	7.52	8.4	8.13	9	0.0181	1.24(0.83 -1.64)	1.72(-0.90 -4.34)
>55 Years	5.71	4.6	5.1	5.56	5.68	4.23	5.34	5.77	7.36	5.8	6.44	6.49	0.0127	1.14 (0.77-1.50)	0.77(-1.19 -2.74)
**Gender**															
Female	6.59	6.06	6.64	7.08	6.23	5.75	6.89	7.35	7.49	8.38	6.81	9.39	0.0052	1.43(1.06-1.79)	2.81(0.67-4.94)
Male	6.22	5.13	4.07	7.41	7.07	5.61	6.67	4.73	7.4	5.62	8.71	5.75	0.1656	0.92(0.48-1.37)	-0.47(-3.41 -2.47)
**Income**															
>$25000	5.73	5.06	5.15	6.03	6.36	4.53	5.26	4.49	5.74	6.21	5.9	6.57	0.1655	1.15(0.83-1.47)	0.84(-0.87-2.56)
<$25,000	7.27	8.48	8.65	9.21	7.33	9.6	9.01	10.64	11.3	11.36	9.43	11.97	0.2776	1.65(0.85-2.45)	4.70 (-0.52 -9.91)
**Race**															
White Non-Hispanic	5.75	4.77	4.35	4.83	6.44	4.75	5.41	4.52	4.93	5.42	5.03	6.73	<.0001	1.17(0.85-1.49)	0.98(-0.76 - 2.73)
Black Non-Hispanic	5.04	7.05	7.19	8.78	6.96	4.17	7.83	8.77	9.7	9.52	10.25	8.93	0.0171	1.77(0.49 - 3.05)	3.89(-1.11 - 8.88)
Multiracial/other race	18.09	8.03	8.14	10.54	9.05	5.26	11.05	14.4	8.52	6.29	12.46	10.23	0.0666	0.57(0.12 -1.01)	-7.87(-19.54 - 3.81)
Hispanic	5	9.91	12.42	15.09	5.29	11.49	12.74	11.3	16.84	13.74	14.87	12.42	0.4613	2.48(0.90 -4.06)	7.41(2.37-12.45)
**Aromatherapy**															
**Day symptoms**															
≤2 days/week	3.56	5.41	6.02	6.02	4.53	5.64	7.11	6.04	9.46	11.41	11.2	12.92	0.0173	3.63(1.08-6.18)	9.36(2.68-16.05)
>2 days/ week	6.19	5.12	5.36	7.12	5.89	5.92	6.99	9.77	10.62	11.33	14.03	13.77	<.0001	2.22(1.47-2.97)	7.58(4.23-10.93)
Throughout the day	4.57	4.75	4.6	4.83	6.32	4.78	4.74	5.08	8.54	7.94	10.71	9.32	<.0001	2.04(1.23 - 2.85)	4.75(2.3 - 7.2)
**Night symptoms**															
≤2 times/ month	11.01	9.29	7.61	7.91	7.89	6.31	6.51	8.41	12.61	14.08	20.89	8.65	0.0048	0.79(0.33-1.24)	-2.37(-8.14 - 3.41)
1–3 times/ week	4.27	7.78	7.76	8.55	7.51	8.91	8.86	8.08	12.1	12.75	13.6	14.63	0.1227	3.42(1.92-4.92)	10.36(6.18-14.53)
≥4 times/week	4.45	3.83	4.02	4.78	5.56	4.23	4.73	5.79	8.25	8.03	10.49	10.14	<.0001	2.28(1.48 - 3.08)	5.69(3.4-7.97)
**Age**															
18-55 Years	6.21	5.98	5.7	6.59	7.41	5.97	6.78	7.43	11.22	11.13	14.21	12.81	<.0001	2.06(1.36-2.76)	6.66(3.63-9.58)
>55 Years	2.48	3.19	3.56	3.54	4.36	3.32	2.97	4.28	5.74	5.93	7.12	7.02	0.0009	2.83(1.67-4.00)	4.55(2.94-6.15)
**Gender**															
Female	5.28	6.48	5.57	5.45	6.55	6.05	6.08	7.43	11.6	11.38	13.69	13.86	<.0001	2.62(1.98-3.27)	8.58(6.07-11.08)
Male	4.17	2	3.69	5.75	5.13	3.26	4.42	4.24	4.73	5.07	7.48	5.11	0.0066	1.23(0.44-2.01)	0.94(-1.86-3.74)
**Income**															
>$25000	4.11	4.1	4.15	4.51	5.3	5.75	4.97	6.12	8.69	8.93	12.91	10.66	<.0001	2.59(1.87-3.32)	6.55(4.49-8.62)
<$25,000	5.05	7.25	7.63	6.38	8.12	6.22	5.36	9.22	7.96	10.26	10.19	8.37	0.5498	1.66(0.80-2.52)	3.33(-0.36 - 7.01)

**Race**															
White Non-Hispanic	4.3	3.92	4.29	4.82	5.64	5	5.41	5.48	9.1	8.92	11.28	11.04	<.0001	2.57(1.89-3.24)	6.74(4.64-8.84)
Black Non-Hispanic	5.47	9.87	6.31	3.8	8.91	4.41	5.48	4.98	4.9	6.31	7.31	6.55	0.636	1.20(0.20-2.20)	1.07(-3.94 - 6.09)
Multiracial/other race	14.65	5.72	9.12	7.31	5.73	5.65	7.35	7.67	8.88	9.04	17.86	11.93	0.0004	0.81(0.15-1.47)	-2.72(-14.07 - 8.63)
Hispanic	2.8	6.45	4.75	9.78	6.79	5.48	5.54	11.62	9.91	9.43	14.4	10.24	0.4036	3.66(1.17-6.15)	7.44(2.66-12.22)
**Yoga**															
**Day symptoms**															
≤2 days/week	3.52	3.81	4.9	6.73	6.9	2.94	4.57	5.11	5.14	4.54	11.31	6.83	0.4274	1.94(0.35-3.53)	3.31(-1.06 -7.69)
>2 days/ week	6.08	5.14	4.26	7.4	6.68	10.66	5.76	5.33	8.51	7.3	8.54	9.66	0.0169	1.59(0.83-2.34)	3.57(-0.42-7.57)
Throughout the day	3.99	5	4.18	5.26	6.12	5.44	4.88	5.13	6.08	6.19	7.78	6.66	0.1088	1.67(1.05 -2.29)	2.67(0.52(4.82)
**Night symptoms**															
≤2 times/ month	6.78	5.78	3.3	5.11	4.71	7.42	5.16	8.66	5.3	7.1	10.34	10.29	0.0061	1.52(0.35-2.68)	3.51(-3.32 -10.33)
1–3 times/ week	6.41	6.32	6.24	6.46	5.22	8.37	7.38	4.99	9.14	6.69	9.5	11.77	0.0567	1.84(0.81-2.86)	5.36(-0.21 -10.93)
≥4 times/week	3.95	4.53	4.03	6.07	6.73	6.27	4.65	4.92	6.25	6.19	7.93	6.43	0.1379	1.63(1.06-2.20)	2.49(0.55-4.42)
**Age**															
18-55 Years	5.01	5.03	4.94	7.51	8.18	8.81	6.24	5.84	7.57	7.65	10.39	9.83	0.0135	1.96(1.29-2.63)	4.81(2.11-7.52
>55 Years	3.44	2.31	3.23	2.77	3.56	3.35	2.6	3.87	4.92	4.12	4.65	3.21	0.0035	0.93(0.54-1.33)	-0.23(-1.65-1.20)
**Gender**															
Female	5.3	6.14	5.28	7.21	8.01	7.89	6.07	5.84	7.12	7.2	10.17	9.62	0.0361	1.82(1.26-2.37)	4.32(1.8-6.84)
Male	3.26	2.46	2.39	3.84	3.2	4.13	3.3	3.87	5.56	4.64	4.57	3.42	0.0481	1.05(0.43-1.67)	0.16(-1.81-2.13)
**Income**															
>$25000	4.61	4.83	4.07	5.46	6.57	7.45	4.83	4.8	6.57	6.63	8.52	6.84	0.0236	1.48(0.93-2.03)	2.23(-0.04-4.49)
<$25,000	4.16	6.07	5.37	6.12	5.56	6.04	5.79	6.59	6.51	5.17	7.97	7.7	0.8746	1.85(0.55-3.16)	3.55(-0.1-8.05)
**Race**															
White Non-Hispanic	4.31	4.38	4.13	5.92	6.21	7.14	5.08	4.55	5.96	6.6	7.06	6.93	0.0421	1.61(1.10-2.11)	2.61(0.76-4.46)
Black Non-Hispanic	2.45	5.68	3.92	3.98	5.67	5.39	4.59	5.19	6.51	3.26	9.61	10.86	0.6097	4.44(0.92-9.79)	8.41(-2.19 - 19.01)
Multiracial/other race	10.85	9.18	5.74	5.96	5.86	5.26	6.74	5.49	10.67	7.7	15	9.57	0.0301	0.88(0.20-1.57)	-1.28(-9.1 -6.54)
Hispanic	4.8	5.33	5.05	7.97	7.81	4.86	4.56	7.28	6.53	4.38	8.36	5.97	0.8887	1.24(0.14-2.35)	1.17(-3.53-5.88)
**Breathing exercises**															
**Day symptoms**															
≤2 days/week	36.8	39.52	37.09	42.06	43.12	39.86	37.46	48.4	43.35	45.2	45.11	45.66	0.165	1.24(0.99-1.49)	8.86(0.30-17.41)
>2 days/ week	37.07	34.45	39.39	39.51	39.97	42.59	36.7	40.62	40.27	45.48	43.04	48.96	0.0009	1.32(1.14-1.50)	11.9(6.11-17.69)
Throughout the day	28.44	27.16	27.84	29.23	31.76	29.8	28.47	30.31	28.51	32.03	35.97	34.21	0.0003	1.20(1.05-1.35)	5.77(1.83-9.71)

**Night Symptoms**															
≤2 times/ month	41.99	40.91	41.65	46.6	39.62	42.91	42.61	52.66	47.47	50.43	52.72	47.41	0.028	1.13(0.86-1.40)	5.42(-5.35 - 16.19)
1–3 times/ week	40.44	39.96	42.99	43.53	42.58	45.49	40.77	45.06	39.83	47.01	46.81	56.55	0.0183	1.40(1.18-1.62)	16.11(8.64-23.59)
≥4 times/week	28.3	27.44	28.07	30.08	32.8	30.45	27.9	31.18	30.1	33.53	35.67	34.71	<.0001	1.23(1.09-1.36)	6.42(2.95-9.88)
**Age**															
18-55 Years	33.28	30.85	33.15	35.19	39.13	36.45	37.83	38.5	36.41	41.15	43.27	43.04	<.0001	1.29(1.14-1.45)	9.77(5.25-14.28)
>55 Years	29.03	27.59	29.48	29.66	26.86	26.41	27.5	27.93	26.83	29.21	30.84	31.36	0.0691	1.08(0.96-1.21)	2.34(-1.16-5.84)
**Gender**															
Female	33.39	33.97	35.08	35.06	37.84	35.92	32.68	36.78	35.15	41.22	41.18	42.58	<.0001	1.28(1.16-1.40)	9.19(5.53-12.86)
Male	28.52	24.35	25.44	30.65	29.61	30.21	28.97	31	28.45	28.13	33.67	31.9	0.0073	1.12(0.92-1.32)	3.38(-1.95-8.71)
**Income**															
>$25000	29.9	28.33	28.59	30.01	35.36	33.09	28.72	32.87	29.74	32.37	37.11	35.72	0.0005	1.19(1.06-1.33)	5.82(2.00-9.64)
<$25,000	37.86	34.93	37.12	36.39	35.77	36.27	37.8	40.14	36.23	40.4	40.74	41.88	0.0522	1.11(0.89-1.32)	4.01(-3.76-11.80)
**Race**															
White Non-Hispanic	30.81	28.66	30.83	32.55	33.34	33.72	30.34	34.22	31.8	36.37	36.78	37.63	<.0001	1.22(1.11-1.33)	6.83(3.66-10.0)
Black Non-Hispanic	40.63	36.65	33.17	35.78	37.53	37.36	36.83	34.23	33.16	40.41	47.77	45.53	0.01	1.12-0.79-1.45)	4.89(-8.00-17.77)
Multiracial/other race	42.37	37.65	41.49	36.46	32.9	39.35	44.01	47.29	35.5	36.66	51.11	43.17	0.09	1.02(0.71-1.33)	0.80(-12.36-13.97)
Hispanic	22.13	35.46	28.62	31.95	39.08	29.15	23.64	30.79	31.58	30.64	31.91	37.39	0.215	1.69(1.11-2.26)	15.24(5.40-25.09)
**Homeopathy**															
**Day symptoms**															
≤2 days/week	2.99	1.66	2.92	0.95	1.57	1.6	1.96	2.74	4.36	2.92	3.49	3.08	0.0039	1.03(0.20-1.87)	0.096(-2.35-2.54)
>2 days/ week	2.91	3.06	2.02	2.85	2.44	1.89	3.31	3.98	4.14	4.53	3.78	5.26	0.003	1.81(0.86-2.76)	2.35(0.19-4.51)
Throughout the day	2.03	2.34	1.94	1.75	1.65	1.91	1.66	2.4	2.3	2.44	3.96	2.97	0.0051	1.46(0.79-2.14)	0.94(-0.25-2.12)
**Night Symptoms**															
≤2 times/ month	4.05	2.63	2.31	1.53	2.64	0.99	1.97	6.74	3.28	5.45	9.33	1.83	0.0086	0.45(0.07-0.84)	-2.22(-5.10-0.66)
1–3 times/ week	2.51	2.1	3.05	2.42	2.3	1.58	3.79	4.36	4.06	4.95	3.99	4.95	0.0076	1.97(0.61-3.34)	2.44(-0.22-5.10)
≥4 times/week	2.11	2.42	1.8	1.89	1.58	2.01	1.81	2.21	2.71	2.43	3.31	3.27	0.0028	1.55(0.91-2.18)	1.12(0.02-2.29)
**Age**															
18-55 Years	2.77	2.71	1.94	2.18	1.94	1.71	2.58	2.65	2.96	3.23	4.39	3.5	0.0005	1.27(0.69-1.85)	0.74(-0.73-2.2)
>55 Years	1.75	1.1	2.16	1.52	2.07	1.24	2.01	3.19	2.82	2.65	2.92	3.55	<.0001	2.03(1.01-3.05)	1.81(0.44-3.17)
**Gender**															
Female	2.75	2.64	2.34	2.25	2.37	2.3	2.24	2.96	3.84	3.13	4.58	4.81	<.0001	1.75(1.1-2.40)	2.06(0.59-3.53)
Male	1.63	2	1.49	1.46	0.85	0.98	1.84	2.62	1.25	2.78	2.44	1.31	0.2711	0.81(0.22-1.39)	-0.32(-1.38-0.75)
**Income**															

>$25000	2.1	2.11	1.98	2.06	2.29	2.32	2.21	2.65	2.75	2.27	3.9	3.12	0.0162	1.48(0.89-2.08)	1.02(-0.05-2.08)
<$25,000	1.9	2.76	1.91	1.44	1.65	0.85	1.7	3.86	2.85	4.82	3.23	5.24	0.0022	2.76(0.68-4.84)	3.34(0.14-6.5)
**Race**															
White Non-Hispanic	2.46	2.01	2.22	2.03	2.02	2.19	2.34	2.72	2.56	2.49	3.27	3.67	0.0012	1.49(0.93-2.06)	1.21(-0.008-2.42)
Black Non-Hispanic	2.84	0.68	1.34	1.17	1.45	0.42	1.03	1.69	1.61	2.04	0.32	2.67	0.0885	0.94(0.14-2.03)	-0.17(-3.35-3.02)
Multiracial/other race	3.6	2.58	2.26	1.73	0.73	2.13	2.69	6.79	1.69	3.04	6.89	2.2	0.0066	0.61(0.13-1.09)	-1.41(-3.66-0.84
Hispanic	0.59	6.49	1.08	2.75	0.95	1.55	1.58	3.05	6.36	5.66	8.46	4.07	0.4592	6.86(1.54-15.26)	3.48(0.71-6.25)
**Naturopathy**															
**Day symptoms**															
≤2 days/week	3.52	1.48	1.69	2.32	1.23	2.31	2.81	2.06	3.38	4.11	2.24	2.48	0.0235	0.70(0.07-1.38)	-0.93(-3.63-1.77)
>2 days/ week	1.86	1.98	1.32	1.5	1.74	2.42	2.3	1.47	3.21	2.2	2.27	3.76	0.0534	2.02(0.76-3.27)	1.89(-0.09-3.87)
Throughout the day	1.19	1.12	1.32	1.07	1.86	1.35	1.49	1.11	1.64	1.78	2.53	1.51	0.0916	1.27(0.57-1.95)	0.31(-0.43-1.06)
**Night Symptoms**															
≤2 times/ month	2.91	1.25	1.99	2.96	1.19	4.26	2.58	4.06	1.85	4.2	3.07	4.54	0.0157	1.56(0.021-3.24)	1.63(-2.29-5.73)
1–3 times/ week	3.08	1.47	1.71	1.04	1.55	2.15	3.05	1.57	4.72	4.11	3.65	3.52	<.0001	1.14(0.08-2.21)	0.44(-2.74-3.61)
≥4 times/week	1.12	1.3	1.22	1.2	1.81	1.37	1.5	1.02	1.74	1.53	2.04	1.77	0.3036	1.58(0.78-2.39)	0.65(-0.11-1.41)
**Age**															
18-55 Years	1.78	1.41	1.4	1.31	2.13	2.6	1.52	1.25	2.47	2.08	2.7	2.35	0.0212	1.32(0.59-2.08)	0.57(-0.60-1.78)
>55 Years	1.43	1.22	1.25	1.2	1.75	1.17	1.8	1.45	1.76	2.08	1.96	1.86	0.0202	1.30(0.58-1.95)	0.43(-0.51-1.30)
**Gender**															
Female	1.86	1.37	1.41	1.31	1.94	2.16	1.82	1.51	2.5	2.49	2.81	2.71	<.0001	1.46(0.78-2.19)	0.85(-0.25-2.02)
Male	1.12	1.45	1.22	1.26	1.41	0.81	1.92	0.98	1.66	1.33	1.71	1.25	0.9246	1.12(0.28-1.84)	0.14(-0.82-0.96)
**Income**															
>$25000	1.11	1	1.22	0.63	1.83	1.99	1.48	1.01	2.06	1.62	2.08	1.73	0.0193	1.56(0.78-2.23)	0.62(-0.13-1.29)
<$25,000	2.2	1.7	2.34	2.08	1.63	1.16	2.62	2.23	2.51	3.05	2.62	4.92	0.0181	2.34(0.27-4.42)	2.82(-0.72-6.35)
**Race**															
White Non-Hispanic	1.59	1.47	1.25	1.4	1.65	1.87	1.75	0.93	2.14	1.92	1.83	2.35	0.045	1.4890.77-2.18)	0.76(-0.24-1.76)
Black Non-Hispanic	1.27	0.55	0.85	0.58	1.37	0.62	0.51	1.42	0.47	1.43	4.23	1.41	0.0263	1.11(0.29-2.50)	0.13(-1.55-1.82)
Multiracial/other race	3.49	1.92	1.89	2.08	0.88	1.69	2.5	3.02	2.24	2.92	4.6	1.26	0.1019	0.36(0.05-0.67)	-2.22(-4.89-0.45)
Hispanic	0.56	1.52	1.94	1.08	2.3	1.31	3.7	2.74	3.59	2.06	2.49	2.15	0.8485	3.82(1.06-8.71)	1.59(-0.11-3.28)
**Vitamins**															
**Day symptoms**															
≤2 days/week	10.16	14.72	8.33	11.06	11.01	10.33	12.28	10.07	11	12.42	11.5	9.28	0.5754	0.91(0.46-1.37)	-0.88(-5.65-3.89)

>2 days/ week	10.05	9.38	8.65	9.6	12.13	9	9.18	10.56	10.6	10.55	6.96	13.97	0.3335	1.39(0.90-1.87)	3.91(-0.53-8.36)
Throughout the day	6.97	7.58	6.91	7.7	7.01	7.48	7.47	7.75	7.34	7.22	7.24	5.5	0.168	0.79(0.56-1.02)	-1.47(-3.27-0.33)
**Night Symptoms**															
≤2 times/ month	13.19	14.08	10.78	12.72	13.11	16.51	15.08	15.13	13.45	17.88	16.25	10.7	0.6415	0.81(0.38-1.25)	-2.48(-8.69-3.73)
1–3 times/ week	13.02	12.3	12.52	11.16	12.1	10.74	11.74	11.09	12.34	14.03	11.28	16.16	0.2573	1.24(0.69-1.79)	3.14(-3.45-9.73)
≥4 times/week	6.45	7.58	6.23	7.48	7.56	6.78	6.99	7.51	7.1	6.85	6.02	6.08	0.0787	0.94(0.69-1.20)	-0.37(-2.05-1.31)
**Age**															
18-55 Years	7.78	9.39	7.36	8.38	10.14	8.57	8.19	8.23	7.47	8.68	6.63	7.86	0.1159	1.01(0.68-1.34)	0.09(-2.44 -2.62)
>55 Years	9.03	9.28	8.24	8.73	6.86	8.67	8.68	9.46	10.13	8.48	8.97	7.72	0.6726	0.85(0.63-1.08)	-1.32(-3.51-0.9)
**Gender**															
Female	8.41	9.48	8.05	8.51	9.03	9.19	8.44	8.86	9.42	8.64	7.28	9.25	0.5347	1.10(0.82-1.38)	0.84(-1.45 -3.130
Male	7.49	7.74	6.69	8.5	8.12	6.07	8.3	8.34	6.83	8.53	7.96	5.36	0.4862	0.72(0.44-0.99)	-2.13(-4.63 - 0.37)
**Income**															
>$25000	7.57	7.36	7.07	6.79	9.24	7.03	6.35	7.51	7.88	7.5	6.37	6.34	0.4764	0.84(0.61-1.07)	-1.22(-3.11(0.66)
<$25,000	9.16	14.08	10.72	11.42	6.89	9.54	10.97	12.15	9.84	12.98	9.25	9.56	0.3052	1.04(0.52-1.56)	0.39(-4.29 -5.08)
**Race**															
White Non-Hispanic	7.23	7.12	6.51	7.7	8.27	8.36	6.66	7.58	7.56	7.84	6.43	8.01	0.8935	1.11(0.83-1.38)	0.78(-1.12-2.72)
Black Non-Hispanic	11.75	12.49	8.91	10.57	8.65	6.93	10.68	6.3	8.92	7.14	9.02	6.37	0.3051	0.5420(0.19-0.89)	-5.38(-11.38 - 0.62
Multiracial/other race	12.21	11.05	9.91	9.29	8	8.22	12.34	13.28	11.08	8.8	6.65	7.52	0.7562	0.62(0.21-1.02)	-4.69(-10.88-1.5)
Hispanic	6.97	18.23	12.35	9.12	12.17	8.27	15.01	14.69	9.96	13.1	12.17	7.98	0.0246	1.14(0.27-2.02)	1.00(-4.61-6.66)
**Acupuncture**															
**Day symptoms**															
≤2 days/week	2.09	0.95	0.33	2.29	0.72	0.91	1.02	2.1	2.86	1.12	7.83	5.34	<.0001	2.56(1.05-6.17)	3.25(-3.12-9.62)
>2 days/ week	1.82	3.21	1.5	1.96	1.91	1.87	0.99	1.23	1.82	1.65	2.19	2.62	0.8624	1.44(0.40-2.48)	0.80(-0.88-2.48)
Throughout the day	1.02	1.91	1.33	1.42	1.22	1.69	1.53	1.02	1.17	1.14	1.88	1.59	0.3408	1.55(0.35-2.75)	0.56(-0.58-1.71)
**Night Symptoms**															
≤2 times/ month	2.78	3.29	0.3	1.42	1.39	0.85	1.46	2.34	3.35	2.02	8.15	1.7	0.0004	0.61(0.01-1.22)	-1.1(-3.44 -1.3)
1–3 times/ week	2.09	2.56	1.43	0.68	1.72	2.16	1.56	1.34	1.81	1.94	4.26	3.14	0.0348	1.50(0.20-2.80)	1.05(-1.26-3.36)
≥4 times/week	1.03	1.98	1.34	1.91	1.29	1.65	1.27	1.03	1.27	1.12	1.63	2.06	0.2133	2.01(0.66-3.36)	1.04(-0.2-2.27)
**Age**															
18-55 Years	1.44	2.89	1.48	1.63	0.82	1.78	1.28	0.95	1.36	1.04	2.2	2.31	0.801	1.61(0.40-2.81)	0.87(-0.72 -2.46)
>55 Years	1.46	1.26	1.09	1.72	2.06	1.55	1.76	1.61	1.65	1.7	2.83	1.93	0.0873	1.32(0.56-2.09)	0.47(-0.51 -1.46)
**Gender**															
Female	1.56	2.6	1.68	2.23	1.27	2.18	1.25	1.23	1.65	1.64	2.83	2.75	0.8369	1.76(0.79-2.73)	1.19(-0.18-2.55)

Male	0.93	1.28	0.5	0.62	1.5	0.62	1.48	1.08	1.15	0.68	1.74	1.17	0.3421	1.26(0.52-3.05)	0.25(-1.34-1.83)
**Income**															
>$25000	1.57	2.46	1.46	2.16	1.72	2.13	1.49	1.3	1.66	1.55	2.22	2.43	0.7503	1.55(0.58-2.52)	0.86(-0.53-2.26)
<$25,000	0.95	1.28	1.08	1.58	0.48	1.58	0.61	1.5	1.24	0.88	4.72	1.46	0.2983	1.54(0.14-2.94)	0.51(-0.60-1.62)
**Race**															
White Non-Hispanic	1.27	1.79	1.32	1.84	1.43	1.93	1.27	0.93	1.5	1.32	1.61	1.86	0.4773	1.46(0.65-2.28)	0.59(-0.35-1.53)
Black Non-Hispanic	0.09	2.58	0.7	1.72	1.8	1.09	1.79	1.1	0.87	0.53	1.89	0.5	0.0004	5.47(3.70-14.64)	0.41(-0.19-1.00)
Multiracial/other race	2.86	2.96	0.79	1.96	0.48	1.02	1.18	1.7	2.02	1.91	5.96	5.22	0.0116	1.83(1.28-4.93)	2.36(-5.72-10.44)
Hispanic	1.87	3.86	1.68	0.6	0.79	0.92	1.21	2.58	1.59	0.9	5.52	3	0.167	1.61(0.74-3.95)	1.13(-2.84-5.10)
**Acupressure**															
**Day symptoms**															
≤2 days/week	2.63	1.46	1.94	1.11	1.86	1.36	0.74	0.79	2.12	3.22	3.63	3.4	0.0065	1.28(0.15-2.70)	0.74(-2.75-4.23)
>2 days/ week	1.7	2.45	1.62	2.17	2	0.62	1.23	1.36	1.85	2.66	2.77	2.34	0.3882	1.37(0.30-2.45)	0.64(-1.08-2.36)
Throughout the day	1.19	1.23	0.96	0.93	1.38	1.22	0.8	1.75	1.11	1.49	1.14	0.75	0.9594	0.63(0.25-1.019)	-0.44(-1.07-0.20)
**Night Symptoms**															
≤2 times/ month	2.32	2.87	1.86	2.81	1.48	0.92	1.1	1.82	1.91	3.9	5.51	2.03	0.3364	0.88(0.30-2.06)	-0.28(-3.11-2.54)
1–3 times/ week	2.47	2.66	2.31	1.42	1.88	0.81	1.8	1.6	1.77	3.56	4.62	3.36	0.0568	1.36(0.08-2.63)	0.88(-2.01-3.77)
≥4 times/week	1.13	1.23	0.95	1.13	1.54	1.14	0.69	1.5	1.24	1.48	0.94	0.99	0.8213	0.87(0.37-1.38)	-0.14(-0.76-0.47)
**Age**															
18-55 Years	1.58	2	1.46	1.28	1.35	0.96	0.63	1.76	1.27	2.2	1.39	1.26	0.8665	0.80(0.26-1.34)	-0.32(-1.27-0.63)
>55 Years	1.4	0.8	1.05	1.21	1.09	1.3	1.26	1.1	1.54	1.49	2.32	1.57	0.0019	1.12(0.45-1.80)	0.17(-0.74-1.08)
**Gender**															
Female	1.38	1.96	1.54	1.45	1.62	1.18	0.97	1.3	1.59	2.38	2.2	1.79	0.4244	1.30(0.64-1.96)	0.41(-0.44-1.26)
Male	1.63	0.83	0.69	0.99	1.47	0.89	0.76	1.94	0.99	1.12	0.92	0.67	0.4304	0.41(0.07-0.75)	-0.96(-2.01-0.01)
**Income**															
>$25000	1.49	1.7	1.27	1.15	1.41	1.46	0.97	1.19	1.42	1.89	1.28	1.18	0.9539	0.80(0.35-1.24)	-0.31(-1.05-0.44)
<$25,000	1.3	1.02	1.61	2.26	2.04	0.38	0.75	2.23	1.04	1.88	1.68	1.11	0.918	0.86(0.20-1.51)	-0.19(-1.11-0.74)
**Race**															
White Non-Hispanic	1.2	1.44	1.25	1.08	1.77	0.95	0.84	0.85	1.62	1.7	1.19	1.31	0.9031	1.09(0.53-1.64)	0.10(-0.55-0.76)
Black Non-Hispanic	0.5	0.52	0.24	1.47	0.78	0.33	1.2	1.06	0.87	0.96	3.56	0.34	0.3915	0.69(0.34-1.72)	-0.15(-0.76-0.46)
Multiracial/other race	3.76	3.12	2.19	2.61	2.27	1.18	2.17	1.82	0.78	3.09	1.66	2.18	0.9283	0.58(0.06-1.10)	-1.58(-4.56-1.40)
Hispanic	2.8	2.28	1.52	1.75	0.15	1.98	0.4	5.9	0.83	2.1	2.34	1.96	0.1008	0.70(0.32-1.72)	-0.83(-4.39-2.73)
**Reflexology**															

**Day symptoms**														
≤2 days/week	1.86	1.9	1.6	2.58	3.01	1.23	0.89	2.39	1.86	2.8	1.23	1.87	0.8004	1.01(0.23-1.79)	0.016(-1.43-1.46)
>2 days/ week	1.88	1.91	1.76	2.28	1.51	1.23	1.28	0.78	1.58	2.1	1.5	1.49	0.665	0.79(0.26-1.33)	-0.39(-1.53-0.75)
Throughout the day	1	1.73	1.29	1.74	1.59	1.61	1.14	0.69	1.43	1.78	0.89	1.25	0.665	1.25(0.45-2.04)	0.25(-0.49-0.99)
**Night Symptoms**														
≤2 times/ month	4.38	3.14	1.27	2.48	4.28	1.31	0.64	2.23	2.88	3.11	2.11	2.79	0.2192	0.64(0.07-1.20)	-1.59(-4.9-1.76)
1–3 times/ week	1.71	2.55	2.44	3.6	1.84	1.24	1.8	1.11	2.04	3.39	2.17	0.99	0.1295	0.58(0.10-1.07)	-0.71(-1.79-0.36)
≥4 times/week	0.95	1.5	1.29	1.57	1.45	1.52	1.05	0.72	1.31	1.59	0.76	1.33	0.0251	1.40(0.63-2.17)	0.38(-0.28-1.03)
**Age**														
18-55 Years	1.19	2.67	1.42	2.24	1.42	1.74	1.11	0.55	1.18	2.01	0.82	1.54	0.0058	1.30(0.51-2.08)	0.35(-0.50-1.21)
>55 Years	1.59	1.09	1.31	1.33	0.89	1.56	0.83	1.51	2.01	1.82	1.44	1.06	0.1152	0.66(0.29-1.04)	-0.53(-1.34-0.27)
**Gender**														
Female	1.35	2.28	1.86	1.68	1.9	1.85	1.27	0.99	1.78	2.5	1.31	1.58	0.048	1.18(0.60-1.76)	0.24(-0.51-0.10)
Male	1.31	0.9	0.7	2.39	1.37	0.74	0.9	0.71	0.99	0.91	0.58	0.97	0.587	0.74(0.12-1.36)	-0.34(-1.31-0.62)
**Income**														
>$25000	1.32	1.48	1.16	1.49	1.16	1.78	1.24	0.84	1.49	2.13	0.92	1.15	0.6011	0.87(0.42-1.33)	-0.17(-0.82-0.48)
<$25,000	1.6	2.01	1.21	2.52	3.86	1.17	0.9	1.26	2.15	2.26	1.75	0.54	0.1078	0.34(0.06-0.62)	-1.06(-2.08- -0.04)
**Race**														
White Non-Hispanic	1.47	1.28	1.51	1.76	1.74	1.68	1.25	0.64	1.42	1.76	1.04	1.38	0.7225	0.94(0.44-1.44)	-0.09(-0.83-0.66)
Black Non-Hispanic	0.33	2.31	1.07	0.69	1.91	1.2	0.72	0.54	0.99	0.77	0.87	1.05	0.6387	3.15(1.20-7.50)	0.72(-0.41-1.85)
Multiracial/other race	2.39	2.11	2.18	4.65	2.43	1.06	1.84	2.62	2.05	2.63	1.99	1.54	0.895	0.644(0.14-1.15)	-0.85(-2.59-0.9)
Hispanic	0.58	4.55	1.37	2.77	2	1.06	0.6	1.99	1.72	2.33	0.98	1.38	0.2378	2.36(0.62-5.34)	0.79(-0.42-2.0)
**Other CAM therapy**														
**Day symptoms**														
≤2 days/week	15.68	8.54	12.85	12.38	12.72	13.03	14.36	11.7	16.34	13.7	12.78	11.79	0.0577	0.75(0.45-1.06)	-3.89(-9.35-1.58)
>2 days/ week	12.94	10.22	9.56	10.82	12.18	11.89	11.62	12.01	10.89	12.91	12.93	13.84	0.0073	1.07(0.75-1.39)	0.90(-3.08-4.87)
Throughout the day	9.16	8.23	8.38	9.96	9.2	5.93	8.99	8.66	7.67	8.3	8.85	8.57	0.7199	0.94(0.72-1.15)	-0.58(-2.64-1.47)
**Night Symptoms**														
≤2 times/ month	19.13	8.65	10.95	10.22	7.91	12.55	11.93	12.25	10.98	19.67	19.74	9.35	<.0001	0.49(0.26-0.72)	-9.78(-16.19- -3.36)
1–3 times/ week	12.25	10.62	12.45	13.31	14.07	9.18	14.97	12.24	10.43	13.39	13.72	10.33	0.996	0.84(0.51-1.17)	-1.92(-6.29-2.45)
≥4 times/week	9.63	8.46	8.02	9.78	9.63	7.05	9.03	9.19	8.93	8.03	8.69	9.93	0.2066	1.03(0.82-1.24)	0.30(-1.71-2.31)
**Age**														
18-55 Years	11.85	10.34	9.42	11.15	10.63	8.97	11.16	10.23	9.88	10.21	10.16	9.71	0.6147	0.82(0.62-1.02)	-2.14(-4.72 -0.43)
>55 Years	8.35	7.03	7.79	8.55	9.34	6.58	8.8	9.01	8.14	9.32	10.15	10.87	0.0014	1.30(1.00-1.60)	2.52(0.29-4.76)

**Gender**														
Female	11.74	9.35	9.66	8.98	10.01	8.25	10.05	10.61	9.56	10.16	10.15	10.12	0.0126	0.86(0.69-1.03)	-1.62(-3.75-0.52)
Male	9.11	7.83	8.02	12.47	10.69	7.68	10.32	8.31	8.6	9.34	10.16	10.2	0.5318	1.12(0.76-1.48)	1.09(-2.03-4.20)
**Income**														
>$25000	10	8.89	8.17	12.29	11.9	7.1	9.37	9.93	10.65	9.3	9.52	10.17	0.4535	1.02(0.80-1.23)	0.17(-1.97-2.31)
<$25,000	10.37	11.09	10.28	8.44	9.75	7.49	11.85	11.52	7.08	9.99	12.8	10.15	0.6127	0.98(0.76-1.18)	-0.21(-4.78-4.33)
**Race**														
White Non-Hispanic	10.44	8.87	8.47	10.39	9.8	8.59	10.1	10.64	9.96	10.53	10.4	10.93	0.0032	1.05(0.86-1.23)	0.48(-1.47-2.43)
Black Non-Hispanic	8.11	7.76	6.37	10.36	11.27	5.32	11.06	5.99	5.86	8.36	6.82	4.92	0.3021	0.61(0.05-1.17)	-3.19(-9.03-2.65)
Multiracial/other race	14.44	9.37	16.9	10.67	10.4	10.35	15.98	9.04	8.14	8.14	12.86	11.1	0.9582	0.77(0.41-1.12)	-3.35(-9.32-2.63)
Hispanic	11.87	9.17	10.63	10.66	12.35	6.69	6.8	8.87	8.72	8.71	9.08	9.2	0.9589	0.78(0.25-1.30)	-2.66(-10.03-4.71)

*Weighted percentages
